# Cloning and characterization of a 9-lipoxygenase gene induced by pathogen attack from *Nicotiana benthamiana *for biotechnological application

**DOI:** 10.1186/1472-6750-11-30

**Published:** 2011-03-30

**Authors:** Fong-Chin Huang, Wilfried Schwab

**Affiliations:** 1 Technische Universität München, Biotechnology of Natural Products, Liesel-Beckmann-Str. 1, D-85354 Freising, Germany

**Keywords:** Lipoxygenase, hydroperoxide lyase, viral vector system, C_9_-aldehyde, 9-hydroxy-10(*E*), 12(*Z*)-octadecadienoic acid (9-HOD), *Nicotiana benthamiana*

## Abstract

**Background:**

Plant lipoxygenases (LOXs) have been proposed to form biologically active compounds both during normal developmental stages such as germination or growth as well as during responses to environmental stress such as wounding or pathogen attack. In our previous study, we found that enzyme activity of endogenous 9-LOX in *Nicotiana benthamiana *was highly induced by agroinfiltration using a tobacco mosaic virus (TMV) based vector system.

**Results:**

A *LOX *gene which is expressed after treatment of the viral vectors was isolated from *Nicotiana benthamiana*. As the encoded LOX has a high amino acid identity to other 9-LOX proteins, the gene was named as *Nb-9-LOX*. It was heterologously expressed in yeast cells and its enzymatic activity was characterized. The yeast cells expressed large quantities of stable 9-LOX (0.9 U ml^-1 ^cell cultures) which can oxygenate linoleic acid resulting in high yields (18 μmol ml^-1 ^cell cultures) of hydroperoxy fatty acid. The product specificity of Nb-9-LOX was examined by incubation of linoleic acid and Nb-9-LOX in combination with a 13-hydroperoxide lyase from watermelon (Cl-13-HPL) or a 9/13-hydroperoxide lyase from melon (Cm-9/13-HPL) and by LC-MS analysis. The result showed that Nb-9-LOX possesses both 9- and 13-LOX specificity, with high predominance for the 9-LOX function. The combination of recombinant Nb-9-LOX and recombinant Cm-9/13-HPL produced large amounts of C_9_-aldehydes (3.3 μmol mg^-1 ^crude protein). The yield of C_9_-aldehydes from linoleic acid was 64%.

**Conclusion:**

The yeast expressed Nb-9-LOX can be used to produce C_9_-aldehydes on a large scale in combination with a *HPL *gene with 9-HPL function, or to effectively produce 9-hydroxy-10(*E*),12(*Z*)-octadecadienoic acid in a biocatalytic process in combination with cysteine as a mild reducing agent.

## Background

Lipoxygenases (LOXs) are nonheme iron-containing enzymes that catalyze the dioxygenation of fatty acids with a 1,4-pentadiene structure and are ubiquitous among eukaryotes [1]. Hydroperoxidation products derived from LOX enzymes can be further converted into other oxylipins through the activity of diverse enzymes downstream in the pathways, including hydroperoxide lyase (HPL), allene oxide synthase, divinyl ether synthase, epoxy alcohol synthase, and peroxygenase [2,3]. These oxylipins include jasmonates, octadecanoids, 6- and 9-carbon aldehydes, oxoacids and divinyl ether fatty acids which are involved in plant defence, senescence, seed germination, plant growth and development.

LOX enzymes can be grouped into two types according to their regiospecificity: 9-LOX, which specifically forms 9-hydroperoxy fatty acid, and 13-LOX, which predominantly catalyzes the formation of 13-hydroperoxy fatty acid. Some LOX enzymes can produce both 9- and 13-hydroperoxy products. Soybeans LOX1, 2, and 3 have different pH optima and different product specificities [4]. LOX1 has a pH optimum of 9.0, producing (13*S*)-hydroperoxy-octadecadienoic acid (HPOD) as the major product from linoleic acid. LOX2 with a pH optimum of 6.1 forms almost equal proportions of 9- and 13-HPODs, whereas LOX3 with a pH optimum of 6.5 produces approximately 65% and 35% 9- and 13-HPODs, respectively [4].

Plant LOXs have been proposed to form biologically active compounds both during normal developmental stages such as germination or growth as well as during responses to environmental stress such as wounding or pathogen attack [1]. Recently, it has been shown that 9-LOX products in plants play an important role in defence responses. In plants, correlative data suggest that 9-LOXs are crucial for lipid peroxidation during the hypersensitive response [5-7]. Pathogen-induced 9-LOX transcript accumulation has been reported in a number of plants, e.g. in tobacco after infection with *Phytophthora parasitica *var. *nicotianae *[8], in potato infected by *Phytophthora infestans *[9], in almond infected by *Aspergillus carbonarius *[10], and in pepper infected by *Xanthomonas campestris *pv *vesicatoria *[11]. Recently, we also found that endogenous 9-LOX activity in *N. benthamiana *was highly induced by agroinfiltration [12]. The strong involvement of 9-LOX in the defence of plants has been demonstrated using antisense strategy or virus-induced gene silencing [8,11]. In addition to having a role in defence responses to pathogens, 9-LOXs also are implicated in plant developmental processes. A specific *9-LOX *gene is transiently induced during potato tuber growth, and its antisense suppression resulted in reduced tuber size [13]. In *Arabidopsis*, 9-*LOX *has been reported to play an important role in late root development [14]. In monocots, maize 9-*LOX *(*ZmLOX3*) has been suggested to be highly involved in regulation of development and to act as a susceptibility factor [15,16].

LOXs also have a role in the production of volatile molecules that can positively or negatively influence the flavour and aroma of many plant products [17]. Volatile C_6_- and C_9_-aldehydes, such as hexanal, (3*Z*)- and (2*E*)-hexenal, (3*Z*)- and (2*E*)-nonenal, as well as (3*Z*,6*Z*)- and (2*E*,6*Z*)-nonadienal are products of unsaturated fatty acids metabolized by LOX and HPL, and are important components of the aroma and flavour of fruits and vegetables. The sequence starts with the oxygenation of linoleic acid and linolenic acid by 9- or 13-LOX to form 9- or 13-hydroperoxy-octadecadienoic/octadecatrienoic acids (HPOD/T), respectively. The 13-hydroperoxy fatty acids can subsequently be cleaved by 13-HPL into 12-oxo-(9*Z*)-dodecenoic acid and hexanal or (3*Z*)-hexenal, whereas the 9-hydroperoxy fatty acids can be cleaved by 9-HPL into 9-oxononanoic acid and (3*Z*)-nonenal or (3*Z*,6*Z*)-nonadienal [18,19]. The (3*Z*)-aldehydes isomerize either spontaneously or enzymatically catalyzed to their (2*E*)-enal isomers and can be reduced to their corresponding alcohols by alcohol dehydrogenase. Due to their organoleptic characteristics, C_6_-aldehydes and alcohols are often called green notes and are widely used as flavours in foods and beverages [20,21]. Besides, (2*E*)-nonenal is considered to be the aged flavour in cereal products, including rice and beer [22,23] whereas (2*E*,6*Z*)-nonadienal was found to have the greatest fresh cucumber odour impact [24,25]. The isomer (3*Z*)-nonenal is used for fresh, tropical, melon notes [21]. (2*E*)-Nonenal and (2*Z*)-nonenal were also identified in Cheddar cheese and were found to play an important role on the odour [26]. Due to their anti-microbial activities, (2*E*)-hexenal, (2*E*)-nonenal and (2*E*,6*Z*)-nonadienal are potential candidates in the control of main mite species in food and feed commodities [27]. These compounds can be extracted from plants or synthesized. However, chemical synthesis is not favoured because consumers have a strong preference for natural food additives [28,29]. In addition, extraction is very expensive because of the low abundance of these short-chain aldehydes and alcohols in plants and cannot meet the increasing market demand for natural flavours. For example, the natural green notes market is estimated at 5-10 ton year^-1 ^and US$ 3000 kg^-1 ^[30]. Therefore, development of a biocatalytic process is required to produce these compounds on a large scale.

Hydroxy fatty acids (HFAs) are multifunctional molecules that have a variety of applications. HFAs and their derivatives are used in cosmetics, paints and coatings, lubricants, and the food industry. HFAs are also encountered in nature as cyclic esters known as lactones which are used in perfumes and as flavour components in food [31,32]. The most important lactone for flavour application with a market volume of several hundred tons per year is γ-decalactone which is transformed from ricinoleic acid (12-hydroxyoctadec-9-enoic acid) [28,33]. Some other examples of HFAs used as precursors of flavour compounds are 13-hydroxy-, and 10-hydroxyoctadecanoic acid and 14-hydroxynonadecanoic acid, from which δ-decanolide, γ-dodecanolide, and γ-nonanolide are generated, respectively [34]. Besides, fatty acids with multiple functional groups can serve as monomers for polymerization and to produce other useful compounds, such as surface active agents [35,36]. Fatty acid hydroperoxides, obtained from LOX action, can act as precursors for further transformation by chemical reactions for the production of HFAs [37,38]. A number of reagents have been used to reduce hydroperoxides into the corresponding hydroxides, such as SnCl_2_, NaBH_4_, KOH, and cysteine [37-39].

We previously demonstrated that 9-LOX activity is highly induced in tobacco cells after treating with viral vectors [12]. In this study, we have isolated the *9-LOX *gene from infiltrated *N. benthamiana *leaves which displayed high 9-LOX activity, and expressed it in yeast cells. The biochemical function of Nb-9-LOX was characterized. The potential biotechnological applications of 9-LOX were described.

## Results

### Expression of the *Nb-9-LOX *gene is induced by pathogen attack but not by wounding

**Figure 1:**
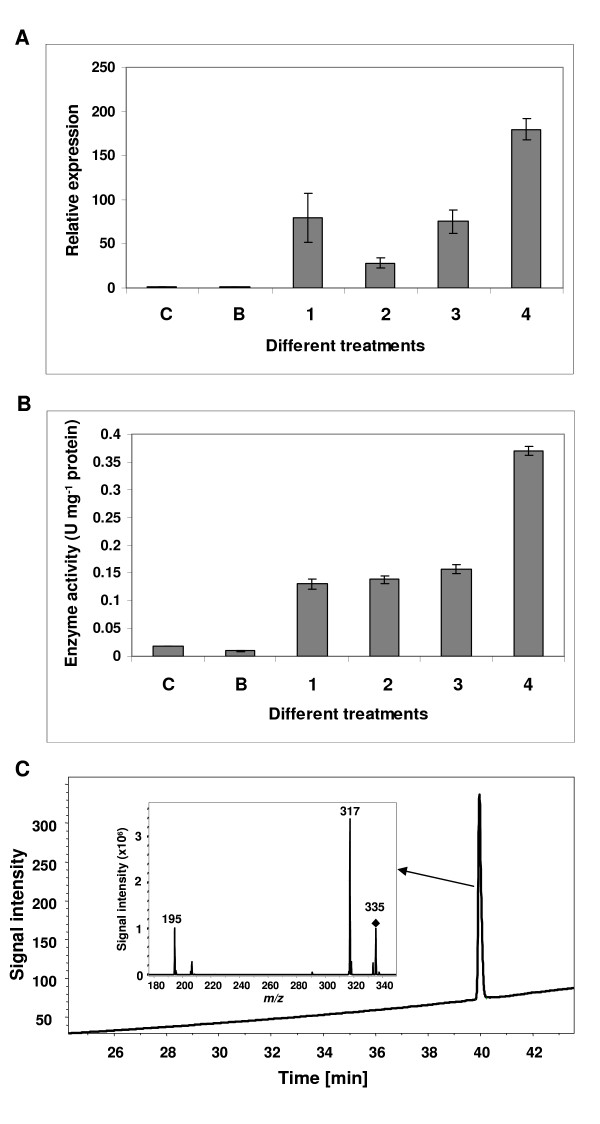
**Analysis of *Nb-9-LOX *gene expression and LOX activity in *N. benthamiana *plants treated with agrobacterial suspensions containing different mixtures of viral provectors**: 1) 3'-provector; 2) 3'-provector and integrase provector; 3) 5'-provector and integrase provector; 4) 3'-provector, 5'-provector and integrase provector; C) untreated *N. benthamiana*; B) leaf infiltrated with buffer. Leaves were harvested 12 days after infiltration. (A) Quantitative real-time RT-PCR analysis was performed using *Nb-9-LOX *and 18S-26S interspacer gene specific primers, the latter used as an internal control for normalization. Values of *Nb-9-LOX *gene expression are means ± SEM of three different evaluations carried out with two sets of cDNAs. (B) LOX activities. LOX activity was measured at pH 7.0 using linoleic acid as a substrate. The reaction products were analyzed by LC-MS. Concentrations of product (9-HPOD) were determined using a standard curve calculated from various known concentrations of 9-HPOD against the UV peak areas which were recorded at 234 nm by LC-MS. Each bar represents the mean and standard error of two replicates. (C) LC-MS analysis of product formed from the reaction containing crude protein extracts of infiltrated tobacco leaf and linoleic acid. 9-HPOD (*m/z *335→195, positive mode) was found to be the main product.

It has been reported that the expression of *LOX *gene(s) is induced by wounding and pathogen infection [5,7,40]. In our previous study, we found that 9-LOX enzyme activity in *N. benthamiana *was highly induced by agroinfiltration using a tobacco mosaic virus (TMV) based vector system [12,41,42]. In order to find out the best material for isolation of the *9-LOX *gene, *N. benthamiana *leaves were infiltrated with a series of mixtures of viral provectors (detailed description see Experimental procedures): 1) 3'-provector; 2) 3'-provector and integrase provector; 3) 3'-provector and 5'- provector; 4) 3'-provector, 5'-provector and integrase provector. The infiltrated leaves were harvested after 12 days and analyzed. The expression levels of *9-LOX *were examined in treated *N. benthamiana *leaves and untreated leaves by real-time PCR using an 18S-26S interspacer gene as an internal control for normalization. As expected, all leaves which were treated with bacterial suspensions showed a much higher expression level of *9-LOX *than untreated leaves (column C) and leaves infiltrated with buffer (column B) (28-180 times) (Figure 1A). Moreover, the expression of *9-LOX *was even more induced, when *N. benthamiana *leaves were co-infiltrated with 3'-provector and 5'-provector together with the integrase provector (column 4). These results revealed that the expression of endogenous *9-LOX *gene in tobacco was more strongly induced by a fully functional RNA replicon. LOX enzyme activities were also monitored in control and infected leaves by LC-MS (Figure 1B). The result showed that 9-HPOD was the main product (Figure 1C). A very low enzymatic activity was detected in the untreated leaves (column C) and leaves infiltrated with buffer (column B). The 9-LOX activities in all infected leaves were significantly higher than those in control leaves. The leaf treated with 3'-provector and 5'-provector together with the integrase provector displayed the highest 9-LOX activity. This result coincided with that of real-time PCR analysis.

**Figure 2:**
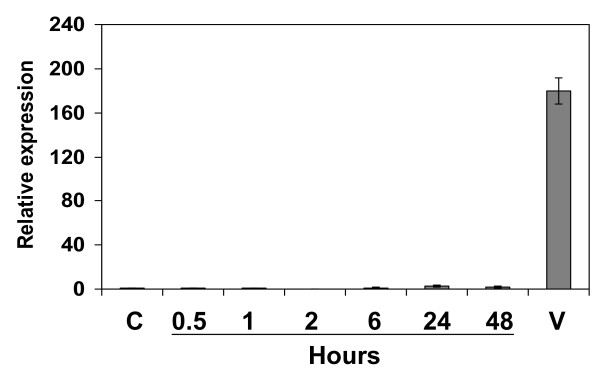
**The effect of wounding on *Nb-9-LOX *gene expression**. Leaves of *N. benthamiana *were infiltrated with buffer and were harvested at 30 min, 1 h, 2 h, 6 h, 24 h, and 48 h after wounding treatment. Quantitative real-time RT-PCR analysis was performed using *Nb-9-LOX *and 18S-26S interspacer gene specific primers, the latter used as internal control for normalization. Values are means ± SEM of three different evaluations carried out with two sets of cDNAs. Column C, untreated *N. benthamiana*; column V, tobacco leaf treated with TMV vectors.

The effect of wounding on *Nb-9-LOX *gene expression was also tested. Leaves of *N. benthamiana *were wounded by infiltration with buffer (detailed description see Experimental procedures). The expression level of *Nb-9-LOX *was analyzed in *N. benthamiana *leaves at different times after wounding by real-time PCR. As a result, gene expression of *Nb-9-LOX *did not show any significant difference after wounding compared to that after infection with the viral vectors (Figure 2). Furthermore, LOX activities were measured in untreated leaf, and leaves at different times after wounding by LC-MS. As expected, 9-LOX activity did not show any clear difference between untreated and wounded leaves (data not shown).

### Cloning and heterologous expression of *Nb-9-LOX *in yeast

For cloning of *9-LOX *gene from *N. benthamiana*, infiltrated leaves with a fully functional RNA replicon which displayed a high LOX activity were chosen according to the induction profiles as described above. The full-length cDNA of *9-LOX *gene was isolated using RT-PCR and a set of primers designed on the basis of a tobacco *9-LOX *gene (Genbank accession number X84040). The cDNA sequence which we obtained from *N. benthamiana *encoded a protein of 862 amino acids with a calculated molecular mass of 97.4 kDa and a predicted pI of 5.52. *N. benthamiana *LOX shows 96% amino acid identity with a previously isolated tobacco 9-LOX [43], and is 87% and 84% identical to a tomato LOX (Genbank accession number AAG21691) and potato tuber LOX (Genbank accession number AAB67865), respectively. We designated this clone as *Nb-9-LOX*.

**Figure 3:**
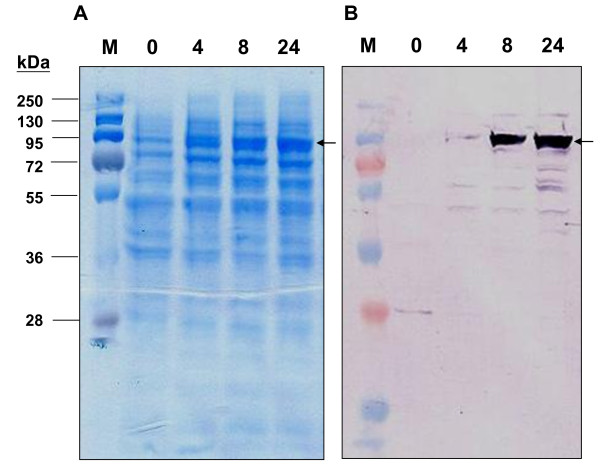
**Western blot analysis**. Total proteins (20 μg) were isolated from yeast cells expressing *Nb-9-LOX *which were harvested at time points corresponding to 0, 4, 8, and 24 hours after galactose induction and separated on a 12% SDS/PAGE gel (A), then transferred to a PVDF membrane and LOX detected with a soybean-LOX antibody (B). The expressed Nb-9-LOX was indicated by arrows (→). M: Marker.

**Figure 4:**
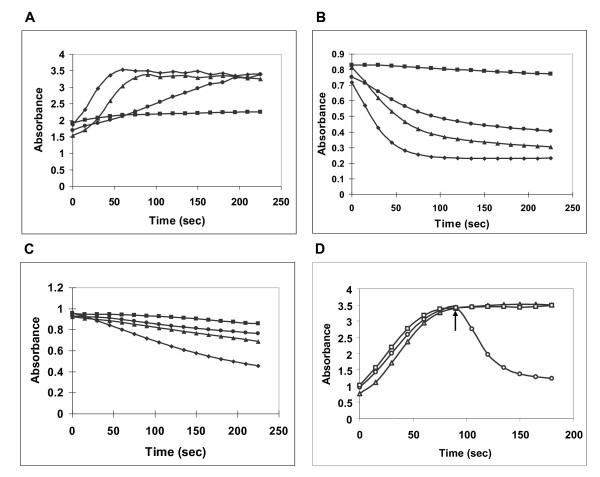
**Analysis of LOX and HPL activities at 234 nm by spectrophotometry**. Total proteins were isolated from yeast cells expressing *Nb-9-LOX *(A), *Cm-9/13-HPL *(B), and *Cl-13-HPL *(C) which were harvested at time points corresponding to 0 (-■-), 4 (-●-), 8 (-▲-), and 24 (-♦-) hours after galactose induction. Nb-9-LOX activity was measured at pH 7.0 using linoleic acid as a substrate, Cm-9/13-HPL activity was measured at pH 7.0 using 9(*S*)-HPOD as a substrate, and Cl-13-HPL activity was measured at pH 6.0 using 13(*S*)-HPOD as a substrate. LOX activity was measured at room temperature by the formation of the conjugated diene at 234 nm, while HPL activity was measured by the decrease of *A*_234 _due to cleavage of the substrate. (D) Reaction of linoleic acid with yeast cell extract of Nb-9-LOX (-Δ-), followed by addition of yeast cell extract of Cl-13-HPL (-□-) or yeast cell extract of Cm-9/13-HPL (-○-) at 90 seconds (arrow).

*Nb-9-LOX *was cloned into a pYES2 vector for expression in yeast to characterize the enzymatic activity of the encoded protein. The crude protein extracts from yeast cells which were harvested at 0, 4, 8, and 24 hours after galactose induction were separated on a 12% SDS-gel. Nb-9-LOX could already be observed on the SDS-gel 4 hours after induction (Figure 3A). Heterologously expressed Nb-9-LOX was identified by Western blot analysis using soybean LOX antibody. The results confirmed that Nb-9-LOX was already expressed 4 hours after induction, and its highest level was observed 24 hours after induction (Figure 3B). LOX activity was measured at room temperature by the formation of the conjugated diene at 234 nm. The crude protein extracts from yeast cells which were harvested at 24 hours after induction displayed the highest LOX activity (Figure 4A). Therefore, Nb-9-LOX used for all assays was prepared from yeast cell culture harvested at 24 hours after induction. Under optimal culture condition, about 0.91 ± 0.13 U LOX activity was obtained from crude protein extracted from 1 ml yeast cell culture. Nb-9-LOX-yeast protein extracts prepared from 1 ml yeast cell culture could convert 18 μmole of linoleic acid into 17.85 μmole of 9-HPOD. The transformation yield was 99% (Table 1).

**Table 1:** Effect of substrate concentration on the yield of 9-HPOD from linoleic acid catalyzed by Nb-9-LOX-yeast protein extracts prepared from 1 ml of cell culture in 20 min.

Linoleic acid (μmol)	9-HPOD (μmol)	Yield (%)
12	11.8 ± 0.4	98
15	14.4 ± 0.6	96
18	17.9 ± 1.5	99
24	20.2 ± 3.0	84
30	19.7 ± 0.7	66

The amount of 9-HPOD was determined using a standard curve calculated from various known concentrations of 9-HPOD against the UV peak areas which were recorded at 234 nm by LC-MS. Each value is the average and standard error of two replicates.

The enzymatic activity of Nb-9-LOX was analyzed at different pH values (pH 2-9) and temperatures (15-45°C) to find out the optimal catalytic conditions,. The yeast-expressed Nb-9-LOX enzyme has a temperature optimum at 35°C with linoleic acid as substrate and a pH optimum of 6.0 with a reproducible slight reduction in activity at pH 7.5, which is near neutrality and similar to that of other 9-LOX enzymes (Additional file 1: Figure S1A and S1B). The *K*_*m *_for the substrate linoleic acid was 3.9 μM (Additional file 1: Figure S1C and S1D).

### Cloning and heterologous expression of *Cm-9/13-HPL and Cl-13-HPL *in yeast

For production of C_6_- or C_9_-aldehydes by a coupled LOX-HPL reaction, full-length cDNAs of *13-HPL *from watermelon leaves (*Cl-13-HPL*, Genbank accession number AY703450) and *9/13-HPL *from melon fruit (*Cm-9/13-HPL*, Genbank accession number AF081955) were isolated using RT-PCR and heterologously expressed in yeast. HPL activity was determined by measuring the decrease of *A*_234 _due to cleavage of the substrate. HPL activities of both Cm-9/13-HPL and Cl-13-HPL could be detected 4 hours after induction, and for both the highest HPL activity was measured 24 hours after induction (Figure 4B and 4C). The activity of Cl-13-HPL was 0.2 ± 0.01 U mg^-1 ^of protein with 13(*S*)-HPOD, while that of Cm-9/13-HPL was 0.94 ± 0.03 U mg^-1 ^of protein with 9(*S*)-HPOD and 1.06 ± 0.08 U mg^-1 ^of protein with 13(*S*)-HPOD. As described in previous studies, Cl-13-HPL has a strong preference for 13-HPODs over 9-HPODs [12,44], and Cm-9/13-HPL has dual activity on both 9- and 13-HPODs [45]. Cm-9/13-HPL cleaves 13-HPOD and 9-HPOD with almost the same efficiency.

### Regiospecificity of Nb-9-LOX

The positional specificity of Nb-9-LOX was determined. At first, Nb-9-LOX was co-assayed with Cm-9/13-HPL or Cl-13-HPL and the absorbance at 234 nm was observed by spectrophotometry. When linoleic acid was incubated with yeast extracts expressing Nb-9-LOX, the absorbance at 234 nm increased rapidly due to the LOX-catalyzed formation of linoleic acid hydroperoxide. After 90 sec, yeast extracts of Cl-13-HPL or Cm-9/13-HPL were added to the reaction mixture. An immediate decrease in absorption at 234 nm (loss of the conjugated diene hydroperoxide) was observed after adding Cm-9/13-HPL yeast extracts (Figure 4D). In contrast, the addition of Cl-13-HPL yeast extracts did not alter absorption at 234 nm. This result revealed the regiospecificity of Nb-9-LOX which specifically forms 9-HPOD, a substrate for 9-HPL.

Furthermore the reaction products formed by Nb-9-LOX were identified by LC-MS analysis. Ion trace at *m/z *195 [C_9_H_16_O_3_+Na]^+ ^was monitored to quantify the production of 9-isomer, whereas for 13-isomer *m/z *247 [C_13_H_20_O_3_+Na]^+ ^was looked at. The result showed that 9-HPOD was mainly produced when Nb-9-LOX-expressed yeast extracts were incubated with linoleic acid. However, 13-HPOD was not detectable (Additional file 1: Figure S2). Under different pH conditions, the product profile was not changed (data not shown). These results suggested that Nb-9-LOX is indeed a 9-specific LOX.

### Production of C_9_-aldehyde in a one-pot process

**Figure 5:**
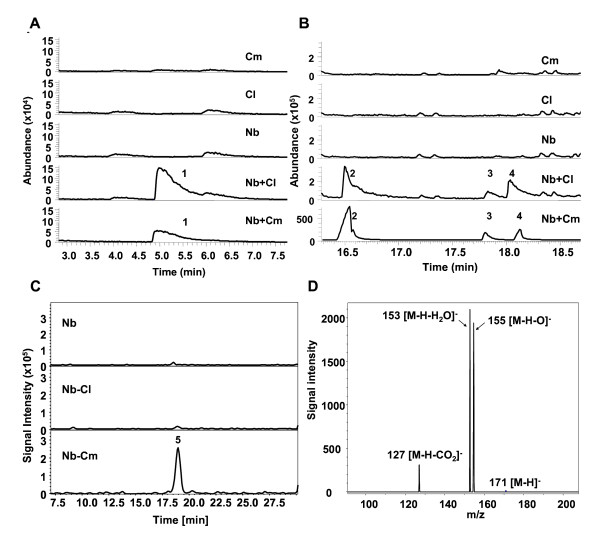
**Analysis of products.** GC-MS analysis of C_6_- (A) and C_9_-aldehydes (B) formed by incubation of linoleic acid and yeast cell crude extracts of Cm-9/13-HPL (Cm), Cl-13-HPL (Cl), Nb-9-LOX (Nb), and Nb-9-LOX in combination with Cl-13-HPL (Nb+Cl) or Cm-9/13-HPL (Nb+Cm). The mass spectra of peaks 1 to 4 yield fragmentation patterns identical to *n*-hexanal (1), (3*Z*)-nonenal (2), putative (2*Z*)-nonenal (3), and (2*E*)-nonenal (4), respectively. (C) LC-MS analysis of the products generated by incubation of linoleic acid with yeast cell extracts of Nb-9-LOX (Nb), Nb-9-LOX and Cl-13-HPL (Nb-Cl), or Nb-9-LOX and Cm-9/13-HPL (Nb-Cm). The product was detected by monitoring *m/z *171. (D) MS/MS spectrum of the [M-H]^- ^ion (*m/z *171) of peak 5. Judging by the fragmentation pattern, peak 5 is assumed to be 9-oxo-nonanoic acid.

The one-pot process is a bioprocess in which the LOX and HPL reactions take place simultaneously. To evaluate the short-chain aldehyde forming activity in the combinations of Nb-9-LOX with Cl-13-HPL or Cm-9/13-HPL, linoleic acid was added to the reaction solutions containing different yeast cell extracts (Figure 5). Reaction products were analyzed by SPME-GC-MS. Low levels of hexanal (*m/z *82, 72, 56, 55) (Figure 5A, peak 1), (3*Z*)-nonenal (*m/z *111, 96, 84, 69, 55) (Figure 5B, peak 2), putative (2*Z*)-nonenal (*m/z *111, 96, 83, 70, 55) (Figure 5B, peak 3) and (2*E*)-nonenal (*m/z *111, 96, 83, 70, 55) (Figure 5B, peak 4) were detected, when linoleic acid was incubated with the crude enzyme solution containing Nb-9-LOX in combination with Cl-13-HPL. A low level of hexanal was also detected in the reaction mixture containing Nb-9-LOX in combination with Cm-9/13-HPL. However, a large amount of (3*Z*)-nonenal, putative (2*Z*)-nonenal, and (2*E*)-nonenal was detected in this reaction (Figure 5A and 5B). Reaction mixtures containing only LOX or HPL enzymes were devoid of C_6_- and C_9_-aldehydes (Figure 5A and 5B). One milligram of crude Cm-9/13-HPL preparation could produce 3317 ± 528 nmol total C_9_-aldehydes in a reaction mixture containing yeast crude extract expressing Nb-9-LOX and linoleic acid after 30 min (Table 2). The yield of C_9_-aldehydes from linoleic acid was 64%.

**Table 2:** Formation of C_6_- and C_9_-aldehydes from linoleic acid in different combinations of LOX and HPL enzymes.

	*n*-Hexanal ** (nmol mg**^**-1 **^protein*)	(3*Z*)-Nonenal ** (nmol mg**^**-1 **^protein*)	(2*Z*)-Nonenal ** (nmol mg**^**-1 **^protein*)	(2*E*)-Nonenal ** (nmol mg**^**-1 **^protein*)
Nb-Cl	48 ± 28	1.9 ± 1.6	0.41 ± 0.37	0.17 ± 0.15
Nb-Cm	30 ± 13	2527 ± 316	360 ± 100	430 ± 143

* Crude extracted protein.

Nb, yeast expressed Nb-9-LOX; Cl, yeast expressed Cl-13-HPL; Cm, yeast expressed Cm-9/13-HPL. The amount of *n*-hexanal and various nonenal isomers were determined using standard curves calculated from various known concentrations of *n*-hexanal and (2*E*)-nonenal against the mass peak areas which were recorded by SPME-GC-MS, respectively. Each value is the average and standard error of three replicates.

The second cleavage product 9-oxo-nonanoic acid formed by 9-HPL activity was analyzed by LC-MS. A large peak in the ion trace *m/z *171 with a retention time of 19.0 min was detected in the reaction mixtures with linoleic acid and Nb-9-LOX in combination with Cm-9/13-HPL (Figure 5C). The MS/MS spectrum of the pseudomolecular ion [M-H]^- ^ion (*m/z *171) showed characteristic ions *m/z *155 [M-H-O]^-^, m/z 153 [M-H-H_2_O]^-^, and *m/z *127 [M-H-CO_2_]^- ^(Figure 5D), and was putatively identified as 9-oxo-nonanoic acid. No major product was detected in the reactions containing Nb-9-LOX alone or Nb-9-LOX in combination with Cl-13-HPL (Figure 5C).

### Biocatalytic hydroxylation of linoleic acid with Nb-9-LOX and cysteine

**Figure 6:**
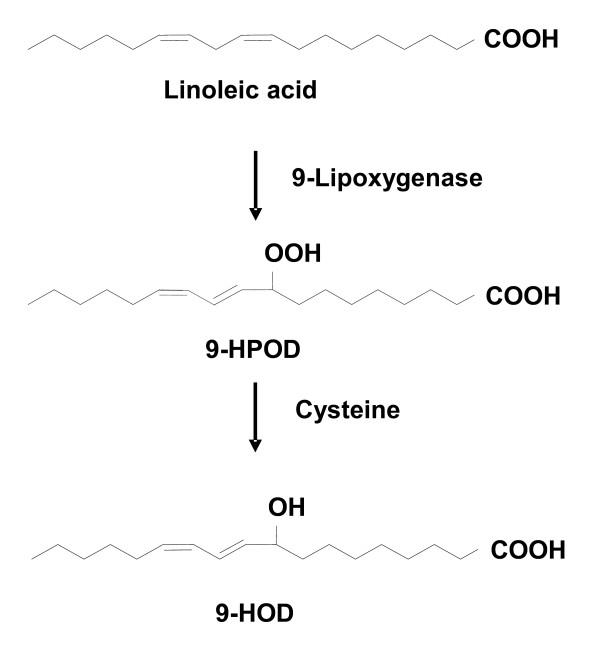
**Scheme for the synthesis of 9-HOD via 9-lipoxygenase and cysteine**. 9-HOD, 9-hydroxy-octadeca-10(*E*),12(*Z*)-dienoic acid; 9-HPOD, 9-hydroperoxy-octadeca-10(*E*),12(*Z*)-dienoic acid.

**Figure 7:**
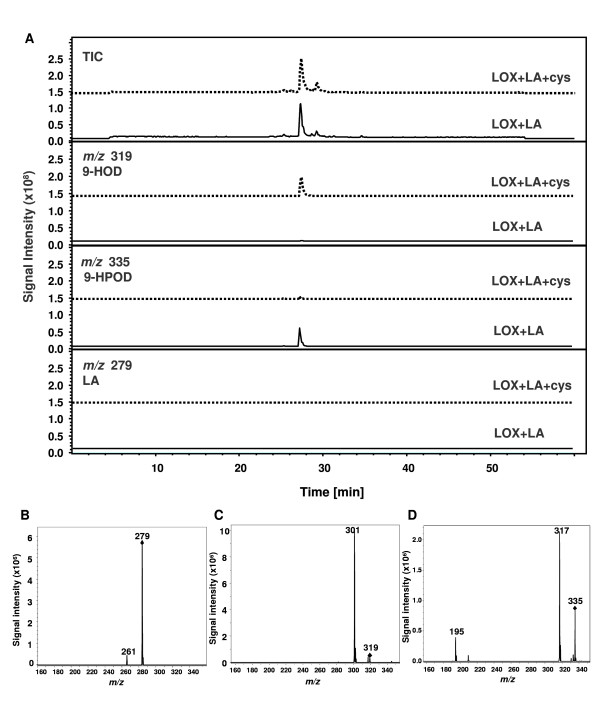
**LC-MS analysis of products generated by incubation of linoleic acid (LA) and yeast cell extracts of Nb-9-LOX (LOX) with or without cysteine (cys)**. (A) Total ion chromatogram (TIC) and ion traces *m/z *319, 335, and 279; 9-HOD, 9-HPOD and linoleic acid were detected by monitoring *m/z *319 [M+Na]^+^, 335 [M+Na]^+ ^and 279 [M-H]^-^, respectively. 9-HOD and 9-HPOD show almost identical retention times but can clearly be distinguished by their MS and MS/MS data. (B) MS/MS spectrum of reference compound linoleic acid (*m/z *279 → 261, negative mode). (C) MS/MS spectum of product 9-HOD (*m/z *319 → 301, positive mode) formed from the reaction LOX+LA+cys from (A). (D) MS/MS spectum of product 9-HPOD (*m/z *335 → 195, positive mode) formed from the reaction LOX+LA from (A).

Fatty acids with multiple functional groups can serve as monomers for polymerization and can be employed to produce other useful compounds [35,36]. In this study, we used a convenient and effective reduction step of hydroperoxides developed by Elshof *et al*. [38] to prepare 9-hydroxy-10(*E*),12(*Z*)-octadecadienoic acid (9-HOD). In this system, the enzymatic large-scale preparation of unsaturated fatty acid hydroperoxides is the first step in the preparation of the corresponding fatty acid hydroxides. A crude extract from yeast cells expressing *Nb-9-LOX *was used as the source of lipoxygenase and cysteine as a mild reducing agent, while the primary substrate was linoleic acid (Figure 6). Products generated by incubation of linoleic acid and yeast cell extracts expressing Nb-9-LOX with cysteine were analyzed by LC-MS. We monitored *m/z *319 [M+Na]^+ ^to detect the production of 9-HOD, whereas for detection of 9-HPOD and linoleic acid *m/z *335 [M+Na]^+ ^and *m/z *279 [M-H]^- ^was monitored, respectively. A large peak at *m/z *319 (9-HOD) was detected when the reaction mixture contained cysteine. Although 9-HOD and 9-HPOD showed almost identical retention times they could clearly be distinguished by their mass spectral data. In contrast, only a tiny amount of 9-HOD was detected in the reaction without cysteine (Figure 7). Moreover, no remaining linoleic acid was detectable in both reactions.

**Figure 8:**
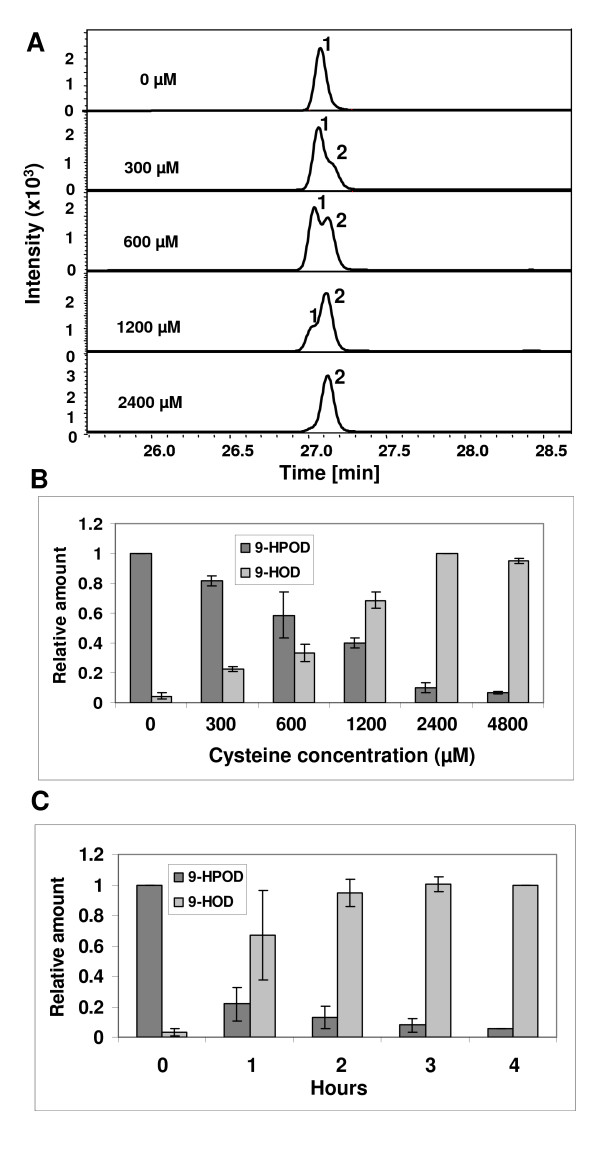
**The effects of cysteine levels and reaction periods on the product profile**. Linoleic acid (600 μM) was incubated with yeast extract expressing Nb-9-LOX and different concentrations of cysteine at 35°C for 1 hour. The product was detected by LC-MS. (A) Ultraviolet detection at 234 nm. Peak 1, 9-HPOD; peak 2, 9-HOD. The concentrations of cysteine are given. (B) Relative amounts of 9-HPOD and 9-HOD calculated from LC-MS analysis of peak 1 (9-HPOD) and peak 2 (9-HOD) from (A). (C) Relative amounts of 9-HPOD and 9-HOD calculated from LC-MS analysis of products formed from the reaction containing 600 μM of linoleic acid, yeast extract expressing Nb-9-LOX, and 2.4 mM of cysteine at 35°C after different reaction periods. The production of 9-HPOD was monitored at *m/z *335 [M+Na]^+^, and of 9-HOD at *m/z *319 [M+Na]^+^. The highest amount was defined as 1. Each bar represents the mean and standard error of three replicates.

In order to develop an optimal system for production of 9-HOD in a biocatalytic process, a series of experiments was performed. At first, the ratio of substrate/cysteine was tested. Linoleic acid (600 μM) was incubated with Nb-9-LOX and different concentrations of cysteine: 300 μM, 600 μM, 1200 μM, 2400 μM, and 4800 μM. Figure 8A shows that the amount of 9-HOD (peak 2) increased with rising levels of cysteine. The best ratio of substrate/cysteine was 1/4 (Figure 8B). A cysteine concentration of more than 4 times that of linoleic acid did not increase the production of 9-HOD. The reaction time was also tested. When the ratio of substrate:cysteine was 1:4, most 9-HPOD was reduced to 9-HOD after two hours (Figure 8C).

For biocatalytic production of 9-HOD, we synchronously added enzyme (Nb-9-LOX-yeast extracts, 0.075 U ml^-1^), linoleic acid (1.5 mM), and cysteine (6 mM) into the bioreactor containing 50 mM citrate buffer, pH 6.0, at 35°C for 2 hrs with gentle shaking at 150 rpm. The products were then extracted with diethylether and analyzed by LC-MS. The result showed that linoleic acid was completely consumed and most 9-HPOD was reduced to 9-HOD (Figure 7). Under this condition, the yield of 9-HOD from linoleic acid was 72% (Table 3). The total amounts of starting material for the production of 1 g of 9-HOD are shown in Table 3. Our system is a very simple batch process, no extensive mixing is required, and no foaming occurs.

**Table 3:** Starting material needed for production of 1 g of 9-HOD.

Material	Amount
Linoleic acid	1.3 g (1.5 mM)
Yeast culture for extraction of Nb-9-LOX	260 ml* (234 units)
Citric acid	33 g (50 mM, pH 6.0)
Cysteine	3.3 g (6 mM)

Yield	72%

* The number was calculated from the data shown in Table 1.

## Discussion

Enhancement of LOX expression in response to fungal, bacterial, and viral pathogen ingress appears to be a general feature occurring both in monocots and dicots. Fournier *et al*. [46] showed that LOX gene expression and activity were induced after root inoculation with zoospores of *Phytophthora parasitica *var. *Nicotinanae *(*Ppn*). The purified LOX from elicited tobacco cells and infected tobacco plants yielded a single band in SDS/PAGE, suggesting that only one LOX isoform might be induced by the pathogen and its elicitors. *In vitro *enzyme assay showed this LOX displayed predominance for the 9-LOX function [46]. Furthermore, a previous study for LOX gene expression in tobacco cell-suspension cultures and intact plants in response to infection with *Ppn *showed that the LOX gene was not constitutively expressed to a detectable level in control cells and healthy plants. In contrast, a rapid and transient accumulation of transcripts occurred in cells and plants after treatment with elicitor and inoculation with zoospores of *Ppn*, respectively [43]. In this work, we also demonstrated that both gene expression and enzyme activity of 9-LOX were induced in *N. benthamiana *leaves treated with agrobacterium suspensions which carried TMV-based vectors. 9-LOX activity was not induced in the leaves after wounding. However, it was induced in all leaves treated with agrobacterium suspension (Figure 1B). LOX gene expression and enzyme activity were even more strongly induced when leaves were treated with agrobacterium suspension carrying 3'-provector, 5'-provector and integrase provector which are assembled in the plant cell to form a fully functional infective RNA replicon. The fully functional RNA replicon is able to replicate autonomously within each infected cell [41,42]. Our data indicate that *Nb-9-LOX *expression is induced by agrobacterium attack and also by TMV infection. The stimulation of LOX activity has also been reported in tobacco after infection with TMV [47]. Infection with TMV has been suggested to result in localized necrotic lesions in hypersensitively reacting tobacco plants [47]. Besides, lipid peroxidation analyses in relation with the hypersensitive reaction in cryptogein-elicited tobacco leaves suggested that 9-fatty acid hydroperoxides are responsible for tissue necrosis [5,7].

Three LOX genes have been isolated from tobacco *Nicotiana attenuata*, namely *NaLOX1*, *NaLOX2*, and *NaLOX3 *[48]. Nb-9-LOX showed 82, 40, and 42% amino acid identity with NaLOX1, NaLOX2, and NaLOX3, respectively. We suggest that Nb-9-LOX has the same function as NaLOX1 because Nb-9-LOX shows a high amino acid identity with NaLOX1, the transcripts of both genes were not detectable in untreated leaf and their transcripts were not induced by wounding. Furthermore, *NaLOX1 *expression is also strongly induced by pathogen infection, and is unlikely to be involved in wounding-induced production of jasmonate [48].

The product specificity of Nb-9-LOX was examined by incubation of linoleic acid and Nb-9-LOX in combination with Cl-13-HPL or Cm-9/13-HPL. The reaction products were analyzed by UV-spectrophotometry, LC-MS and SPME-GC-MS. Figure 4D shows that the absorbance did not decrease when Cl-13-HPL was added to the reaction containing Nb-9-LOX and linoleic acid, indicating that the hydroperoxide produced by Nb-9-LOX from linoleic acid was not cleaved by Cl-13-HPL. In contrast, the addition of Cm-9/13-HPL caused a decrease of absorbance. The reaction product formed by incubation of linoleic acid with Nb-9-LOX was analyzed by LC-MS. 9-HPOD was the main hydroperoxide product, while 13-HPOD was not detectable (Figure 2S). The result from SPME-GC-MC showed that low levels of C_6_-aldehyde and C_9_-aldehyde were produced during the reaction of linoleic acid with recombinant Nb-9-LOX in combination with recombinant Cl-13-HPL (Figure 5). This result suggested that Nb-9-LOX functions like 13-LOX to some small extent. The low level of 13-HPOD was further metabolized into hexanal by Cl-13-HPL. As a large amount of 9-hydroperoxide substrates is formed in the reaction, a detectable level of C_9_-aldehydes can be produced in spite of low activity of Cl-13-HPL toward 9-hydroperoxides [12,44]. Likewise, the low level of 13-HPOD was further metabolized into a small amount of hexanal by Cm-9/13-HPL. However, the high level of 9-hydroperoxide was converted into a large amount of C_9_-aldehydes by Cm-9/13-HPL which displayed high 9-HPL activity (Figure 5). The results of SPME-GC-MS revealed that Nb-9-LOX possesses high 9-LOX specificity with a tiny share of 13-LOX activity, although 13-product was not detected by LC-MS. This result could also explain why a low level of C_6_-aldehydes and C_9_-aldehydes were detected when fatty acids were incubated with *Cl-HPL *treated leaf extracts (displayed high activity of both 9-LOX and 13-HPL) as described previously [12].

Chemical synthesis is the easiest way to produce large amounts of C_6_- or C_9_- aldehydes and alcohols. However, for food application, consumers have a strong preference for naturally synthesized additives and aromas. Due to the high demand for such natural flavours, many groups have attempted to develop a biocatalytic process to produce these compounds on a large scale. During the past years, enzymatic syntheses using LOX and HPL as biocatalysts have become popular for large scale production of C_6_-aldehydes [28]. For this purpose, production of stable LOX and HPL with high enzymatic activity is the first step. Heterologous gene expression in plants, yeast cells, or bacteria would be an excellent method to increase the availability of LOX and HPL for that purpose. Different sources of HPLs have been explored for industrial production of C_6_-aldehydes using soybean flour as a source of stable LOX. For example, a recombinant alfalfa 13-HPL expressed in *E. coli *in combination with soybean LOX resulted in yields of 50% for hexanal and 26% for hexenal from vegetable oils [49]. A combination of watermelon 13-HPL-overexpressing tobacco leaf tissue and soybean LOX2 yielded 50% of hexenals from linolenic acid [50]. A combination of HPL isolated from green bell pepper and soybean LOX1 isolated from defatted soybean meal resulted in 37% yield for the hexenal isomers from linseed oil [51]. In addition, a yield of 60% for (3*Z*)-hexenal from linolenic acid was obtained via the combination of a 13-HPL isolated from sugar beet leaves and soybean LOX1 [52]. Besides, a combination of watermelon 13-HPL-overexpression tobacco leaf tissues and soybean VLXC expressed in yeast yielded 93% hexanal from linoleic acid [12]. However, up to now no study has described the production of C_9_-aldehydes on a large scale. In this study, we isolated a *9-LOX *gene responding to pathogen attack from *N. benthamiana *leaves. We have successfully expressed this LOX gene (*Nb-9-LOX*) together with a *9-HPL *(*Cm-9/13-HPL*) in yeast cells. The yeast cells expressed 0.9 U ml^-1 ^and 0.1 U ml^-1 ^cell cultures of stable 9-LOX and 9-HPL, respectively. Our results showed that the combination of recombinant Nb-9-LOX and recombinant Cm-9/13-HPL could produce large amounts of C_9_-aldehydes. The yield was 64% for nonenal isomers together from linoleic acid (Table 2). Only a very low level of C_6_-aldehyde was formed (about 1/100 of that of C_9_-aldehydes, Table 2). Therefore, this system has potential for producing C_9 _compounds on a large scale.

A number of reagents have been used to reduce hydroperoxides into the corresponding hydroxides [37-39]. However, not all of them are suitable and economically attractive for a biocatalytic process. Cysteine is a mild reducing agent which has been used to effectively reduce hydroperoxides into the corresponding hydroxides in a bioprocess [38]. Based on this, we tried to produce 9-HOD using yeast expressed Nb-9-LOX and cysteine. Cell lysates containing 9-LOX activity can be easily and rapidly prepared from yeast cells using glass beads. In this system, the enzymatic large-scale preparation of unsaturated fatty acid hydroperoxides is the first step in the preparation of the corresponding fatty acid hydroxides. Large-scale conversion of fatty acids into hydroperoxides by soybean LOX has been successfully accomplished [53-55]. In order to obtain high yields of hydroperoxides, some points have to be taken into account: the optimal enzyme/substrate ratio, the substrate concentration, and adequate oxygen supply. At high substrate concentration, fatty acids form aggregates which are not easily dispersible in buffer [53]. A low oxygen concentration may eventually lead to an anaerobic reaction resulting in unwanted side-products [54]. Besides, Elshof *et al. *[38] pointed out that higher conversion yields could be obtained with gradual additions of substrate and enzyme. For obtaining high yields of hydroxides, also the optimal substrate/cysteine ratio and time point of adding of cysteine to the incubation mixture have to be considered [38]. In our system, we synchronously added enzyme (Nb-9-LOX-yeast crude extracts), linoleic acid, and cysteine into the bioreactor. The dioxygenation of linoleic acids by yeast expressed Nb-9-LOX resulted in high yields of hydroperoxy fatty acids. The use of the mild reducing agent cysteine makes it simple and efficient to produce 9-HOD. The biocatalytic process described here allows rapid and cost-efficient generation of HFAs in one experimental step. The procedure is simple because it requires no solvent or surfactant, and is conducted at atmospheric pressure.

## Conclusions

We have isolated a *9-LOX *gene from *N. benthamiana*, which is induced by pathogen attack. This LOX gene could be expressed in yeast cells in stable and large amounts. It efficiently transforms linoleic acid to 9-HPOD. This LOX gene can be used to produce C_9_-aldehydes in combination with a *HPL *gene with 9-HPL function, or to produce 9-HOD in a biocatalytic process in combination with cysteine as a mild reducing agent.

## Methods

### Chemicals

Chemicals used were standard commercial products of analytical grade from the following companies: linoleic acid (Roth, Karlsruhe, Germany); 9(*S*)-HPOD and 13(*S*)-HPOD (Biozol Diagnostica, Eching, Germany); cysteine (Sigma, Steinheim, Germany); citric acid monohydrate (Merck, Darmstadt, Germany).

### Wounding treatment of leaves

For investigation of effect of wounding on *Nb-9-LOX *gene expression, leaves of *N. benthamiana *were infiltrated with buffer (10 mM 2-*N*-morpholino-ethanesulfonic acid (MES) pH 5.5, 10 mM MgSO_4_) and were harvested at 30 min, 1 h, 2 h, 6 h, 24 h, and 48 h after wounding.

### Viral vectors and agroinfiltration

The viral vector system based on cr-TMV (crucifer-infecting tobacco mosaic virus) is an expression system that relies on *in planta *assembly of functional viral vectors from separated pro-vector modules [41,42]. The 5' module (pICH17388) contains the 5' part of the viral vector including the RNA-dependent RNA polymerase, movement protein genes, the coat protein subgenomic promoter, and a *loxP* site. The 3' module (pICH11599) contains a *loxP* site, cloning sites for cloning of the gene of interest (unused in the present study), and the 3' end of the viral vector. Both modules are assembled inside a plant cell with the help of a site-specific recombinase (pICH14011) to form a fully functional RNA replicon.

Agrobacterium was used to deliver various modules into plant cells. pICH17388, pICH14011, and pICH11599 were separately transformed into the *Agrobacterium tumefaciens *strain AGL0 using the freeze-thaw technique as described by Höfgen and Willmitzer [56], and integrity was confirmed by PCR. *Agrobacterium *strains carrying each pro-vector module were mixed and infiltrated into *N. benthamiana *using a syringe without a needle as described [12].

### Real-time RT-PCR analysis

Total RNA was extracted from leaves of transfected *N. benthamiana *and untreated control plants using the CTAB extraction procedure [57]. RNA samples were treated with RNase free DNase I (Fermentas, St. Leon-Rot, Germany) for 1 h at 37°C. First strand cDNA synthesis was performed in duplicate in a 20 μl reaction volume, with 1 μg of total RNA as the template, random primer (random hexamer, 100 pmol), and M-MLV reverse transcriptase (200 U, Invitrogen, Karlsruhe, Germany) according to the manufacturer's instructions. Real-time PCR was performed as described by Huang *et al*.[12]. A relative quantification of gene expression was performed using an 18S-26S interspacer gene as a reference [58]. Primers for the amplification of 18S-26S interspacer gene were 5'-ACC GTT GAT TCG CAC AAT TGG TCA TCG-3' (forward) and 5'-TAC TGC GGG TCG GCA ATC GGA CG-3' (reverse). The primers used for the target gene *Nb-9-LOX *were 5'-ATA TGT GCC AAG GGA CGA-3' (forward) and 5'-AAT AGG CCT TCG CCA TCA-3' (reverse). Relative expression ratio was calculated and normalized using an 18S-26S interspacer gene [58].

### Cloning of full length cDNAs of *Nb-9-LOX*, *Cl-13-HPL *and *Cm-9/13-HPL*

Total RNA was isolated from leaves of *N. benthamiana *treated with viral vectors, leaves of watermelon (*Citrullus lanatus*), and fruit of melon (*Cucumis melo*) by CTAB extraction [57]. The first-strand cDNAs were synthesized from 10 μg of total RNA using Superscript III RTase (Invitrogen, Karlsruhe, Germany) and a GeneRacer oligo-dT primer (5'-GCT GTC AAC GAT ACG CTA CGT AAC GGC ATG ACA GTG T_(18)_-3').

The coding regions of *Nb-9-LOX*, *Cl-13-HPL*, and *Cm-9/13-HPL *were amplified by RT-PCR with the corresponding cDNA template prepared as described above. The primers were Nb-9-LOX-S and Nb-9-LOX-AS (Table 4, design based on a tobacco *9-LOX *gene, accession number X84040) for *Nb-9-LOX*, ClHPL-S and ClHPL-AS (Table 4, design based on a watermelon *HPL *gene, accession number AY703450) for *Cl-13-HPL*, and CmHPL-S and CmHPL-AS (Table 4, design based on a melon *HPL *gene, accession number AF081955) for *Cm-9/13-HPL*. The temperature program used was 5 min at 95°C, 1 cycle; 45 sec at 95°C, 45 sec at 55°C, 2 min at 72°C, 35 cycles; final extension at 72°C for 10 min. The PCR products amplified with Phusion enzyme polymerase (New England Biolabs, Frankfurt, Germany) were A-tailed with Taq-DNA polymerase and ligated into the pGEM-T vector (Promega, Mannheim, Germany). The recombinant genes were subjected to sequencing to confirm the sequence of the inserts.

**Table 4:** Primer sequences used for PCR amplification of coding regions of *LOX *and *HPL *genes for cloning into the pYES2 vector.

Genes	Sequences	Cloning sites
*Nb-9-LOX*	Forward: 5'-CGGGGTACCAACACAATGTCTCTGGAGAAGATT-3'	*Kpn*I/*Not*I
	Reverse: 5'- ATTGCGGCCGCCTATATTGACACACTGTT-3'	
*Cm-9/13-HPL*	Forward: 5'- CGCGGATCCTACACAATGTCTACTCCTTCTTCC-3'	*Bam*HI/*Xho*I
	Reverse: 5'- CCGCTCGAGTTAAACCATATCGGTTGC-3'	
*Cl-13-HPL*	Forward: 5'- CGGGGTACCAACACAATGAAGGTCACCATGACC-3'	*Kpn*I/*Not*I
	Reverse: 5'- ATTGCGGCCGCTCAGTTGGTCCTTTGAAA-3'	

The underlined nucleotide sequences indicate the sites of the restriction enzymes for insertion into multiple cloning sites of a plasmid.

### Expression of *Nb-9-LOX*, *Cl-13-HPL*, and *Cm-9/13HPL *in yeast

The full-length open reading frames of *Nb-9-LOX*, *Cl-13-HPL*, and *Cm-9/13-HPL *were excised from pGEM-T vectors (constructed as described above), and cloned into pYES2 vectors (Invitrogen, Karlsruhe, Germany) to generate pYES2-*Nb-9-LOX*, pYES2-*Cl-13-HPL*, and pYES2-*Cm-9/13-HPL*. Furthermore, these three constructs were transformed into the *S. cerevisiae *INVSc1 strain for expression of recombinant protein as described [12]. Time-course studies of *Nb-9-LOX*, *Cl-13-HPL*, and *Cm-9/13-HPL *gene expression in yeast were performed by harvesting an aliquot of cells at 0, 4, 8, and 24 hours after galactose induction.

### SDS/PAGE and western blot analysis

Western blot analysis was performed to detect the recombinant Nb-9-LOX in yeast. Total proteins (20 μg) were separated on a 12% Tris-glycine SDS/PAGE gel (Anamed, Groß-Bieberau, Germany), and then electrophoretically transferred onto a PVDF membrane (Roth, Karlsruhe, Germany). The Nb-9-LOX protein was detected with a polyclonal rabbit anti-LOX antibody (product number: AS06 128, Agrisera, Vännäs, Sweden) as described by Huang *et al*. [12].

### Enzyme extraction and assay

For analysis of the LOX activity in tobacco leaves, one hundred milligram (fresh weight) samples of *N. benthamiana *leaves infiltrated with *Agrobacterium *were ground into a fine powder in liquid nitrogen with a mortar and a pestle, followed by being resuspended in 300 μl of protein extraction buffer (50 mM sodium phosphate buffer, pH 7.5, 10 mM EDTA, 0.1% Triton X-100, 5 mM β-mercaptoethanol). The homogenate was centrifuged at 4°C, 16,000 × g for 10 min to remove the cell debris. Total protein content was determined by Bradford assay. LOX activity was determined in 500 μl of 50 mM sodium phosphate buffer (pH 7.0) containing 3 μl of *N. benthamiana *leaf extracts and 600 μM of linoleic acid at 25°C with constant shaking for 30 min. The reaction products were extracted with chloroform/methanol (2:1, v/v), evaporated to dryness, resuspended in 30% methanol, and analyzed by LC-MS as described by Huang *et al*. [12].

For analysis of LOX and HPL activities in yeast extracts, yeast cells were harvested by centrifugation and resuspended in a volume of breaking buffer (50 mM sodium phosphate buffer, pH 7.5, 1 mM EDTA, 5% glycerol, 1 mM PMSF) to obtain an OD_600 _of 50. Cell lysates were prepared using glass beads by vortexing mixture for 30 seconds, followed by 30 seconds on ice. The procedure was repeated ten times for a total of ten minutes. Cell debris was removed by centrifugation (5000 g, 5 min, 4°C). Total protein content was determined by Bradford assay. LOX activity was measured at room temperature by the formation of the conjugated diene at 234 nm. Yeast cell extracts (1 μl) were added to 120 μl of 50 mM sodium phosphate buffer containing 625 μM of linoleic acid and measured spectrophotometrically at 234 nm applying an extinction coefficient of 23000 M^-1 ^cm^-1^. Initially, LOX activity was measured at pH 7.0, after determination of the pH optimum pH 6.0 was used. HPL activity was determined in 120 μl of 50 mM sodium phosphate buffer (pH 6.0 for Cl-13-HPL, and pH 7.0 for Cm-9/13-HPL) containing 2.5 μl of yeast cell extracts and 50 μM of substrate (9(*S*)-HPOD or 13(*S*)-HPOD) at room temperature. The decrease of fatty acid hydroperoxide was measured spectrophotometrically by following the decrease of *A*_234 _due to cleavage of the substrate by hydroperoxide lyase. The concentration of remaining substrate was calculated using an extinction coefficient of 23000 M^-1 ^cm^-1^. One unit of activity (U) corresponds to the amount of enzyme that converts 1 μmol of substrate per minute.

For pH optimum determination, 50 mM citric acid was used for pH range of 4-6, 50 mM phosphate buffer for pH range of 6-8 and 50 mM Tris buffer for pH range of 8-9.

### Product identification

For analysis of aldehyde formation in a one-pot LOX-HPL process, 10 μl of yeast extract of Nb-9-LOX in combination with 50 μl of yeast extract of Cl-13-HPL or Cm-9/13-HPL were diluted to 2 ml with 50 mM sodium phosphate buffer (pH 7.0) containing 0.15 mM linoleic acid. The mixture was incubated for 30 min at 25°C with constant shaking in a 20 ml reaction vial closed with a septum. Headspace compounds were trapped by SPME (65 μm polydimethylsiloxane-divinylbenzene coated fibre, Supelco, Steinheim, Germany) at 45°C for 30 min. Subsequently, the SPME fibre was introduced into the GC injector and thermally desorbed volatiles analyzed by MS [12]. Diagnostic ions for hexanal were *m/z *72 and 82, whereas *m/z *69 and 83 were used for nonenal. The amount of *n*-hexanal and various nonenal isomers were determined using standard curves calculated from various known concentrations of *n*-hexanal and (2*E*)-nonenal against the mass peak areas which were recorded by SPME-GC-MS, respectively.

For analysis of non-volatile reaction products formed in a one-pot LOX-HPL process, 10 μl yeast extract of Nb-9-LOX and 40 μl yeast extract of Cm-9/13-HPL or of Cl-13-HPL were added to 500 μl of 50 mM sodium phosphate buffer (pH 7.0) containing 600 μM linoleic acid at 25°C with constant shaking for 1 hour. The reaction products were extracted with chloroform/methanol (2:1, v/v), evaporated to dryness, resuspended in 30% methanol, and analyzed by LC-MS. The HPLC system consisted of a quaternary pump and a variable wavelength detector, all from Agilent 1100 (Bruker Daltonics, Bremen, Germany). The column was a LUNA C18 100A 150 × 2 mm (Phenomenex, Aschaffenburg, Germany). HPLC was performed with the following binary gradient system: solvent A, water with 0.1% formic acid and solvent B, 100% methanol with 0.1% formic acid. The gradient program was as follows: 0-10 min, 70% A/30% B to 50% A/50% B; 10-40 min, 50% A/50% B to 100% B, hold for 7 min; 100% B to 70% A/30% B, in 3 min, then hold for 10 min. The flow rate was 0.2 ml/min. Absorbances were recorded at 234 nm for the detection of hydroperoxy fatty acids (9- and 13-HPOD). Amounts of 9-HPOD were determined using a standard curve calculated from various known concentrations of 9-HPOD against the UV peak areas which were recorded at 234 nm. The production of 9-HPOD was monitored at *m/z *195 [C_9_H_16_O_3_+Na]^+^, whereas for 13-HPOD *m/z *247 [C_13_H_20_O_3_+Na]^+ ^was monitored in positive mode. 9-Oxo-nonanoic acid was monitored at *m/z *171 [M-H]^- ^in negative mode.

For analysis of reaction products formed by Nb-9-LOX and cysteine from linoleic acid, the products were partitioned into diethylether, the solution was concentrated, dissolved in 30% methanol and analyzed by LC-MS. Absorbances were recorded at 234 nm for the detection of 9-HPOD and 9-HOD. The production of 9-HOD was monitored at *m/z *319 [M+Na]^+^, whereas for detection of 9-HPOD *m/z *335 [M+Na]^+ ^was monitored. Linoleic acid was monitored at *m/z *279 [M-H]^-^. Concentrations of 9-HOD were determined using a standard curve calculated from various known concentrations of 9-HPOD against the UV peak areas which were recorded at 234 nm by LC-MS.

## Authors' contributions

FCH isolated the *9-LOX *gene, produced and characterised the recombinant protein, performed the qPCR and LC-MS analyses, and drafted the manuscript. WS conceived the study, and participated in its design and coordination. All authors read and approved the final manuscript.

## Note

Accession numbers: tobacco 9-LOX (accession number X84040), Cl-13-HPL (accession number AY703450), and Cm-9/13-HPL (accession number AF081955).

## Supplementary Material

Additional file 1 **Properties of Nb-9-LOX and LC-MS analysis of products formed by Nb-9-LOX**. Temperature optimum, pH optimum, determination of Nb-9-LOX kinetic constants, determination of *K*_*m *_value and LC-MS analysis of hydroperoxy fatty acids (HPOD) formed from linoleic acid catalyzed by Nb-9-LOX Click here for file
