# Supplementary material for: Cloning and characterization of a 9-lipoxygenase gene induced by pathogen attack from Nicotiana benthamiana for biotechnological application
Source: BMC Biotechnol. 2011 Mar 30;11:30. doi: 10.1186/1472-6750-11-30 (PMC3079629; doi:10.1186/1472-6750-11-30)
Supplement: Additional file 1 — Properties of Nb-9-LOX and LC-MS analysis of products formed by Nb-9-LOX. Temperature optimum, pH optimum, determination of Nb-9-LOX kinetic constants, determination of Km value and LC-MS analysis of hydroperoxy fatty acids (HPOD) formed from linoleic acid catalyzed by Nb-9-LOX [file 1472-6750-11-30-S1.DOC]

**Fig. S1.**

1. Temperature optimum of Nb-9-LOX activity. Values are the means of two separate determinations performed using Nb-9-LOX-expressed yeast protein extracts in 50 mM Na-phosphate buffer (pH 7.0) with 600 μM linoleic acid. The formation of 9-HPOD was analyzed by HPLC. The optimal temperature for Nb-9-LOX activity is indicated by a vertical dashed line.
2. pH optimum of Nb-9-LOX activity. Values are the means of three separate determinations performed using Nb-9-LOX-expressed yeast protein extracts in 50 mM citrate (-■-), phosphate (-●-), or Tris-HCl (-▲-) buffer mixture with 50 μM linoleic acid. LOX activity was measured at room temperature by the formation of the conjugated diene at 234 nm. The optimal pH value for Nb-9-LOX activity is indicated by a vertical dashed line.
3. Determination of Nb-9-LOX kinetic constants for linoleic acid. Values are the means of three separate determinations performed using Nb-9-LOX-expressed yeast protein extracts in 50 mM citrate buffer (pH 6.0) with different concentration of linoleic acid. LOX activity was measured at room temperature by the formation of the conjugated diene at 234 nm.
4. Determination of *K_m_* using the Lineweaver-Burke plot.

**Fig. S2.** LC-MS analysis of hydroperoxy fatty acids (HPOD) formed from linoleic acid (LA) catalyzed by Nb-9-LOX. The production of 9-isomer was monitored at *m/z* 195 [C_9_H_16_O_3_+Na]^+^, whereas for 13-isomer *m/z* 247 [C_13_H_20_O_3_+Na]^+^ was monitored.
